# Psychometric evaluation of the Chinese version of the resilience scale for parents of children with cancer

**DOI:** 10.3389/fpsyg.2024.1378639

**Published:** 2024-07-18

**Authors:** Gaoxiang Zhong, Zhangyi Ding, Xichen Li, Yan Qiao, Xinmiao Zhang, Huixia Cui

**Affiliations:** ^1^Department of Nursing, Jinzhou Medical University, Jinzhou, China; ^2^School of Nursing, Wannan Medical College, Wuhu, China

**Keywords:** childhood cancer, parents, resilience, reliability, validity, psychometric properties

## Abstract

**Background:**

Pediatric cancer ranks among the leading causes of mortality in children globally. While serving as primary caregivers, certain parents may experience anxiety, depression, and other related challenges. However, not all parents succumb to such psychological distress. Resilience emerges as a potential protective factor. Assessing parental resilience holds paramount importance for healthcare professionals in identifying issues and offering tailored interventions. Yet, mainland China lacks adequate tools for this assessment. Hence, this study endeavors to translate the Resilience Scale for Parents of Children with Cancer (RSP-CC) into Chinese and scrutinize its psychometric properties.

**Methods:**

From April 2023 to January 2024, a methodological study was conducted in Chengdu, Chongqing, and Jinzhou, China, recruiting 377 eligible parents via convenience sampling for a multicenter cross-sectional survey. The translation process of the RSP-CC adhered rigorously to the Brislin model, involving forward and back-translation, followed by necessary modifications. Item analysis was assessed using the critical ratio and the item-total correlation coefficient. Validity evaluation encompassed content and internal validity assessments. Scale reliability was determined through Cronbach’s α coefficient, retest reliability, and split-half reliability coefficients.

**Results:**

The Chinese version of the RSP-CC comprises 4 dimensions and 24 items, explaining a cumulative variance contribution of 63.58%. In this investigation, the content validity index attained a score of 0.97. Exploratory factor analysis (EFA) yielded four factors consistent with the original scale, while confirmatory factor analysis (CFA) indicated satisfactory fit indices. Both Cronbach’s α coefficient and retest reliability stood at 0.95, with a split-half reliability coefficient of 0.82.

**Conclusion:**

After rigorous translation and verification, the RSP-CC was adapted in China, demonstrating favorable psychometric characteristics. It stands as an effective instrument for parents of children diagnosed with cancer in China. Additionally, this scale could serve as a crucial tool for clinical staff in formulating specific interventions.

## 1 Introduction

Pediatric cancer stands as a leading cause of mortality among children globally, imposing significant burdens on both the afflicted child and their family (Lubega et al., 2021). Global Cancer Statistics indicate an annual diagnosis of approximately 380,000 children worldwide (Sung et al., 2021), with nearly 90% of cases occurring in low to middle-income countries (Cayrol et al., 2022). China, as a developing nation, grapples with a substantial pediatric cancer burden, too. World Health Organization data reveals that nearly 120,000 children in China were diagnosed with cancer between 2015 and 2020, comprising 14% of global juvenile cancer incidences (Ji et al., 2022).

In China, prevalent types of childhood cancer include leukemia, brain and central nervous system malignancies, and lymphomas (Ni et al., 2022). The diagnosis of cancer can inflict profound physical, psychological, social, and behavioral distress upon both the child and their primary family caregivers (Cheng et al., 2023). Throughout the arduous journey of childhood cancer treatment, parents assume the primary caregiving role due to their child’s tender age. Confronted with the treatment’s intricacies and the burden of severe symptoms, parents endure heightened levels of stress compared to their children (Law et al., 2019). Nevertheless, research indicates that despite adversities, a substantial portion of parents actively engage in their child’s treatment, participate in decision-making, and provide both material and emotional support (Van Schoors et al., 2015). Within this context, parental resilience emerges as a pivotal factor.

Resilience denotes an individual’s capacity to adapt effectively when confronted with adversity, trauma, tragedy, threat, or severe stressors (Bajjani-Gebara et al., 2019). Chung et al. (2023) demonstrated a positive correlation between parental resilience in the context of childhood cancer and coping strategies (*r* = 0.53), while revealing negative correlations with anxiety (*r* = −0.57) and depression (*r* = −0.42). Similarly, Mohammadsalehi et al. (2022) found a positive association between parental resilience and self-efficacy (*r* = 0.37). Parents with high resilience may tend to exhibit superior coping skills and a more positive outlook, facilitating their ability to navigate stressors and adversities effectively. Conversely, parents with low resilience may find themselves more vulnerable to the challenges posed by their child’s cancer diagnosis, with potential repercussions on their quality of life and psychological well-being (Da Silva et al., 2010; Luo et al., 2022). Consequently, bolstering parental resilience emerges as a key strategy for alleviating psychological distress and enhancing overall quality of life. Recognizing the significance of this issue, Prof. Önal (Önal et al., 2023) developed the RSP-CC to assess the resilience of parents. The scale has 4 dimensions and 24 items, with acceptable results in terms of reliability, content validity, and internal validity (Önal et al., 2023).

Currently, the Conner-Davidson Resilience Scale stands as the predominant tool for gaging resilience levels in China (Connor and Davidson, 2003). However, its applicability is limited to general outpatients, psychiatric patients, and those with anxiety disorders, rendering it unsuitable for the specific population of parents of children with cancer. Utilizing this scale in such a context may compromise the validity of the results. Hence, there is a pressing need for a dynamic, multidimensional, and validated Chinese version of the RSP-CC tailored to assess parental resilience and evaluate the efficacy of pertinent interventions.

According to the definition of Patterson’s Family Resilience Theory (Patterson, 2002), factors affecting resilience are generally categorized into protective and risk factors. When experiencing a major crisis, protective and risk factors interact and mutually influence each other. In this study, coping and social support represent protective factors, whereas emotional stress and caregiver burden constitute risk factors. Chronic negative emotions can impede parental coping mechanisms (Koutelekos et al., 2023), while social support has been shown to alleviate such emotions, whereas caregiver burden exacerbates them (Guralnick et al., 2008; Güven Baysal and Çorabay, 2024). Moreover, the presence of social support can mitigate caregiver burden (Adib-Hajbaghery and Ahmadi, 2019). These four factors dynamically interact to influence a parent’s resilience. The purpose of this study was to translate the RSP-CC into Chinese following the Brislin translation model and to assess its psychometric properties through item analysis, content validity analysis, internal validity analysis, internal structural validity analysis, and reliability analysis.

## 2 Materials and methods

### 2.1 Study design and participants

This study adopted a methodological approach. Following comprehensive training and approval from relevant hospitals, two clinicians and one clinical nurse from the Cancer Hospitals of Chengdu, Chongqing, and Jinzhou, China, conducted participant recruitment from April 2023 to January 2024. Inclusion criteria comprised parents of children aged ≤18 years diagnosed with cancer via pathological investigation, serving as primary caregivers, and providing informed consent for research participation. Exclusion criteria encompassed parents of children with additional severe or chronic illnesses, parents of children who received palliative treatment, and parents with communication or cognitive impairments, as determined by language proficiency and cognitive functioning assessments. This study was conducted under the authorization of Prof. Önal and obtained endorsement from the Ethics Committee of Jinzhou Medical University (Ethics Approval No. JZMULL2023129). Prior to survey administration, parents received clear explanations regarding the survey’s objectives, significance, and precautions.

Participant sample size was determined in accordance with factor analysis guidelines. Exploratory factor analysis (EFA) necessitates samples five to ten times the total number of scale items (Knapp and Sawilowsky, 2004), while confirmatory factor analysis (CFA) requires a minimum of 200 cases (Lasmarías et al., 2021). Considering different sample sources for EFA and CFA, and accounting for a 10% invalid questionnaire rate, a minimum of 352 cases was deemed necessary for inclusion. In this study, 400 questionnaires were distributed, with 385 recovered, and 377 deemed valid, yielding a valid recovery rate of 94.25%. Questionnaire completion time averaged 8–10 min, with data collected onsite. Subsequently, a follow-up survey was conducted with a randomly selected subset of 50 parents after a two-week interval to assess retest reliability.

### 2.2 Instruments

#### 2.2.1 The general demographic characteristics questionnaire

After conducting a thorough review of prior research and consulting with experts, a tailored questionnaire was developed to collect demographic data from both parents and children. The questionnaire encompassed details such as parents’ gender, age, educational background, occupation, economic status, as well as the child’s age, gender, diagnosis, and treatment stage.

#### 2.2.2 The resilience scale for parents of children with cancer

The degree of resilience in parents of children with cancer was assessed using the RSP-CC. This scale comprises 4 dimensions and 24 items: coping (12 items), emotional stress (5 items), social support (4 items), and caregiver burden (3 items) (Önal et al., 2023). Responses were measured on a 5-point Likert scale, ranging from “strongly disagree” (1) to “strongly agree” (5), with a total score range of 24–120, where higher scores indicate greater resilience. This scale was validated by 601 parents of children with cancer in Turkey, and the results showed that the overall Cronbach’s α coefficient was 0.99 (Önal et al., 2023).

### 2.3 Translation and revision of the scale

Following authorization from Prof. Önal, the translation and refinement of the RSP-CC were meticulously conducted in accordance with the Brislin model (Brislin, 1970). Initially, the scale was translated into Chinese by a Ph.D. candidate in oncology proficient in English, alongside an English professor with overseas experience. Subsequently, forward translations were rendered into English by a nursing specialist and a foreign language instructor from the UK, respectively, forming reverse translation versions. Finally, two proficient bilingual specialists compared and deliberated on the translations and the original scale, culminating in the development of a draft Chinese version of the RSP-CC.

A panel of seven experts (comprising two pediatricians, two psychologists, and three oncologists) was convened to refine the draft RSP-CC. Selection criteria mandated a minimum of 6 years of relevant research in pediatric oncology psychology, attainment of at least an attending physician level, and possession of a master’s degree. The expert panel consisted of 5 males and 2 females, with 3 holding master’s degrees and 4 doctorates, and averaging (18.14 ± 6.01) years of professional experience.

After cross-cultural adaptation, a preliminary investigation involving 30 parents was conducted utilizing the revised RSP-CC. This phase aimed to gather feedback on scale comprehension, content relevance, and emotional resonance. Among the participants, one mother suggested revising item 7 from “Having cancer in my life has made me a more understanding and tolerant person” to “In my life, the presence of my child’s cancer has made me a more understanding and tolerant person.” Additionally, she proposed changing item 13 from “The advent of cancer in my life has caused me to become an anxious person” to “In my life, the appearance of my child’s cancer has turned me into an anxious person” to provide a clearer definition of cancer and better alignment with the reading preferences of Chinese people. Similarly, a father suggested changing item 15 from “I have not been sleeping as well since cancer entered my life” to “I have not slept as well since my child was diagnosed with cancer.” The research team reviewed and incorporated these recommendations, and finally, the Chinese version of the RSP-CC was developed.

### 2.4 Data analysis

Data analysis was performed using AMOS 24.0 and SPSS 26.0. Continuous data were expressed as mean ± standard deviation, while categorical data were presented as frequency and percentage (%). Significance was established at *p* < 0.05.

#### 2.4.1 Item analysis

Item analyses were evaluated by using the critical ratio and correlation coefficient methods. The critical ratio was utilized to assess item differentiation, with a ratio of ≥3 considered indicative of appropriate differentiation (Raykov and Marcoulides, 2016). Additionally, homogeneity was evaluated through calculation of the item-factor correlation coefficient, with a coefficient of ≥0.40 indicating satisfactory homogeneity (Raykov and Marcoulides, 2016).

#### 2.4.2 Content validity analysis

Seven qualified specialists were engaged in evaluating the relevance of the items for this study. The content validity index of each item (I-CVI) was computed by dividing the number of specialists rating an item with 3 or 4 points by the total number of participating specialists. The content validity index of the scale (S-CVI) was determined by averaging the I-CVI values across all items. Generally, when I-CVI ≥ 0.78 and S-CVI ≥ 0.90, the scale’s content validity is considered satisfactory (Zamanzadeh et al., 2015).

#### 2.4.3 Internal validity analysis

In this study, the internal validity of the Chinese version of the RSP-CC was assessed by exploratory factor analysis (EFA) and confirmatory factor analysis (CFA). The collected samples were randomly divided into two subsamples. Subsample 1 (*n* = 177) was used to conduct EFA, and subsample 2 (*n* = 200) was used to conduct CFA.

The EFA was used to delineate the underlying structure of the scale factors (Schreiber, 2021). If the value of the Kaiser-Meyer-Olkin (KMO) is more than 0.60 and Bartlett’s test of sphericity yields a significant result, it indicates that EFA could be conducted (Tobias and Carlson, 1969). The factors were extracted based on the principal axis factoring (PAF), the principle of eigenvalue >1, and the promax rotation method. Four conditions must be met: (1) all of the factor loadings remain greater than 0.40, (2) the absolute value of the difference between the two factor loadings should be at least greater than 0.20, (3) at least three of the extracted items should be attributed to one factor, and (4) the cumulative variance contribution should be >0.40 (Schreiber, 2021).

The CFA was conducted to further validate the rationality of the model’s factor structure. To make the results more robust, the data were analyzed for multivariate normality before choosing the estimation method for CFA. The results indicated a *p*-value of 0.14 (>0.05), suggesting that the data were multivariate normally distributed. Based on this result, a structural equation model was constructed using the maximum likelihood ratio method, with items as observed variables and factors as latent variables for CFA. In this four-factor model, coping, emotional stress, social support, and caregiver burden encompass the primary psychological and social challenges encountered by parents of children with cancer (Önal et al., 2023). These factors interact and mutually influence each other. By comprehensively studying these four factors, healthcare workers can gain insight into the parents’ psychological adaptation process, laying the groundwork for developing tailored psychological interventions and support measures. The specific numerical requirements are as follows: (1) chi-square degree of freedom (χ^2^/ν) < 3, (2) root mean square residual (RMSR) < 0.05, (3) Tucker-Lewis index (TLI), comparative fit indices (CFI), and incremental fit indices (IFI) > 0.90, and (4) root mean square error of approximation (RMSEA) < 0.08. In general, when the RMSEA value exceeds 0.08, it suggests a poor model fit; when the RMSEA falls within the range of 0.05–0.08, it indicates an acceptable model fit; and when the RMSEA is below 0.05, it signifies a good model fit (Shi et al., 2022).

#### 2.4.4 Internal structure validity analysis

The convergent and discriminant validity were evaluated by calculating the average variance extracted (AVE), the combined reliability (CR), and the square root of the AVE. The standards for evaluation are as follows: the AVE must be >0.50, the CR must be >0.70, and the square root of the AVE must exceed the correlation coefficients between the relevant factors (Fornell and Larcker, 1981).

#### 2.4.5 Reliability analysis

The split-half reliability coefficient and Cronbach’s α coefficient were calculated to assess internal consistency. Subsequently, 50 parents previously identified were reevaluated using the same scale after 2 weeks to determine retest reliability. It is widely acknowledged that for a scale to exhibit good reliability, these indexes must all exceed 0.70 (Chang et al., 2018).

## 3 Results

### 3.1 The general demographic characteristics of parents

This study involved a total sample of 377 parents, comprising 120 fathers (31.8%) and 257 mothers (68.2%). Among them, 228 (60.5%) were aged between 31 and 40, with an average age of (35.19 ± 5.94) years. Furthermore, 123 (32.6%) had graduated from junior high school, 80 (21.1%) were unemployed, 124 (32.9%) reported a family income ranging from 2,001 to 4,000 yuan per month, and 164 (43.5%) resided in urban areas. Detailed information is available in Table 1.

**Table 1:** The general demographic characteristics of parents.

Characteristic	Group	Total sample (*n* = 377) *n* (%)	Subsample 1 (*n* = 177) *n* (%)	Subsample 2 (*n* = 200) *n* (%)
Gender	Male	120 (31.8)	67 (37.9)	53 (26.5)
	Female	257 (68.2)	110 (62.1)	147 (73.5)
Age	≤30	86 (22.8)	34 (19.2)	52 (26.0)
	31–40	228 (60.5)	121 (68.4)	107 (53.5)
	41–50	56 (14.9)	19 (10.7)	37 (18.5)
	>50	7 (1.9)	3 (1.7)	4 (2.0)
Education level	Elementary school and below	16 (4.2)	9 (5.1)	7 (3.5)
	Junior high school	123 (32.6)	87 (49.2)	36 (18.0)
	Senior high school	70 (18.6)	15 (8.5)	55 (27.5)
	Junior college	96 (25.5)	37 (20.9)	59 (29.5)
	Undergraduate and above	72 (19.1)	29 (16.4)	43 (21.5)
Occupation	Farmer	68 (18.0)	27 (15.3)	41 (20.5)
	Worker	86 (22.8)	48 (27.1)	38 (19.0)
	Employees of public institutions	79 (21.0)	33 (18.6)	46 (23.0)
	Self-employed	53 (14.1)	19 (10.7)	34 (17.0)
	Unemployed	80 (21.2)	44 (24.9)	36 (18.0)
	Others	11 (2.9)	6 (3.4)	5 (2.5)
Site	Chengdu City	174 (46.2)	77 (43.5)	97 (48.5)
	Chongqing City	121 (32.1)	43 (24.3)	78 (39.0)
	Jinzhou City	82 (21.8)	57 (32.2)	25 (12.5)
Economic situation	≤2,000 yuan	57 (15.1)	28 (15.8)	29 (14.5)
	2,001–4,000 yuan	124 (32.9)	54 (30.5)	70 (35.0)
	4,001–6,000 yuan	81 (21.5)	27 (15.3)	54 (27.0)
	6,001–8,000 yuan	54 (14.3)	25 (14.1)	29 (14.5)
	8,001–10,000 yuan	38 (10.1)	25 (14.1)	13 (6.5)
	>10,000 yuan	23 (6.1)	18 (10.2)	5 (2.5)
Location	Rural	146 (38.7)	68 (38.4)	78 (39.0)
	Town	67 (17.8)	32 (18.1)	35 (17.5)
	Urban	164 (43.5)	77 (43.5)	87 (43.5)

### 3.2 The general demographic characteristics of children

Regarding the children of participating parents, 241 (63.9%) were male, with 199 (52.8%) aged ≤6 years and an average age of (6.72 ± 4.39) years. Additionally, 145 (38.5%) had acute lymphoblastic leukemia, 72 (19.1%) had other types of leukemia, and 63 (16.7%) had lymphoma. Among them, 286 (75.9%) were undergoing current treatment. Detailed information is provided in Table 2.

**Table 2:** The general demographic characteristics of children.

Characteristic	Group	Total sample (*n* = 377) *n* (%)	Subsample 1 (*n* = 177) *n* (%)	Subsample 2 (*n* = 200) *n* (%)
Gender	Male	241 (63.9)	126 (71.2)	115 (57.5)
	Female	136 (36.1)	51 (28.8)	85 (42.5)
Age	≤6	199 (52.8)	87 (49.2)	112 (56.0)
	7–12	129 (34.2)	62 (35.0)	67 (33.5)
	13–18	49 (15.0)	28 (15.8)	21 (10.5)
Disease diagnosis	Acute lymphoblastic leukemia	145 (38.5)	62 (35.0)	83 (41.5)
	Other types of leukemia	72 (19.1)	41 (23.2)	31 (15.5)
	Lymphomas	63 (16.7)	33 (18.6)	30 (15.0)
	CNS tumors	12 (3.2)	8 (4.5)	4 (2.0)
	Sympathetic nervous tumors	23 (6.1)	8 (4.5)	15 (7.5)
	Malignant Bone Tumors	16 (4.2)	6 (3.4)	10 (5.0)
	Soft-tissue sarcoma	26 (6.9)	11 (6.2)	15 (7.5)
	Other solid tumors	20 (5.3)	8 (4.5)	12 (6.0)
Treatment stage	Early stage of diagnostics	21 (5.6)	9 (5.1)	12 (6.0)
	Treatment period	286 (75.9)	135 (76.3)	151 (75.5)
	Relapse and return to hospital	52 (13.8)	23 (13.0)	29 (14.5)
	Review and follow up	18 (4.8)	10 (5.6)	8 (4.0)

### 3.3 Items analysis

The results of the item analysis revealed critical ratios (t-values) ranging from 10.59 to 22.47, indicating a high level of item differentiation within the scale. Moreover, the correlation coefficients between individual item scores and their corresponding factor scores ranged from 0.71 to 0.89, indicating a high degree of homogeneity among the items of the scale. Furthermore, the Cronbach′s α coefficient of the corresponding factor decreased after deleting any of the item, so all 24 items could be retained. For detailed information, please see Table 3.

**Table 3:** Item analysis of the scale.

Factor	Item	Item score (SD)	Critical ratio	Item-factor correlation coefficient	*p*	Cronbach’s α of factor	Cronbach’s α of factor if item deleted
Factor 1	1	2.297 (0.949)	21.840	0.851	0.026	0.945	0.938
	2	2.745 (1.013)	19.011	0.785	0.003		0.941
	3	2.454 (0.886)	20.806	0.793	0.011		0.940
	4	2.393 (0.841)	18.410	0.777	0.017		0.940
	5	2.440 (0.877)	19.744	0.811	0.001		0.939
	6	2.406 (0.895)	18.044	0.780	0.007		0.940
	7	2.475 (0.841)	15.353	0.706	0.036		0.943
	8	2.334 (0.850)	18.365	0.805	0.005		0.939
	9	2.332 (0.862)	19.745	0.778	0.006		0.940
	10	2.350 (0.942)	22.468	0.838	0.009		0.938
	11	2.435 (0.882)	17.242	0.753	0.015		0.941
	12	2.462 (0.905)	18.784	0.790	0.003		0.940
Factor 2	13	2.249 (0.915)	13.280	0.885	0.030	0.897	0.859
	14	2.260 (0.885)	12.527	0.841	0.015		0.874
	15	2.172 (0.914)	10.585	0.828	0.002		0.880
	16	2.286 (0.877)	12.362	0.822	0.011		0.880
	17	2.305 (0.887)	13.372	0.830	0.014		0.878
Factor 3	18	2.488 (0.866)	15.621	0.842	0.031	0.884	0.861
	19	2.565 (0.867)	17.311	0.838	0.004		0.863
	20	2.764 (0.925)	17.312	0.889	0.012		0.835
	21	2.462 (0.922)	18.531	0.875	0.007		0.844
Factor 4	22	2.268 (0.847)	12.311	0.727	0.004	0.914	0.867
	23	2.233 (0.907)	15.316	0.831	0.028		0.865
	24	2.220 (0.918)	13.763	0.795	0.013		0.897

### 3.4 Content validity analysis

In this study, a total of seven experts meeting the specified criteria were enlisted to assess the items of the RSP-CC. The results revealed I-CVI values ranging from 0.86 to 1.00, with an S-CVI of 0.97.

### 3.5 Internal validity analysis

**Figure 1:**
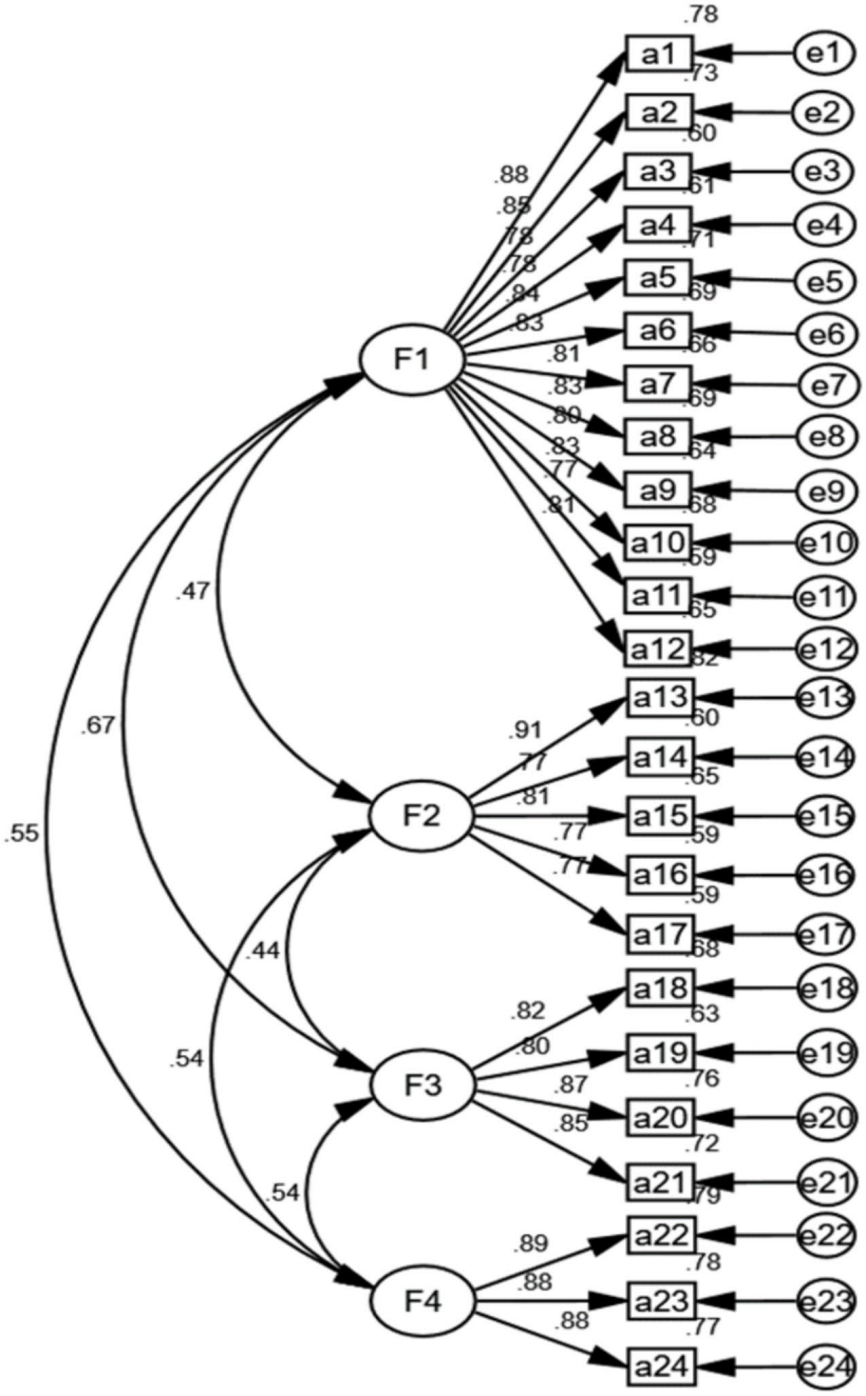
Standardized four-factor model of the.

The EFA results showed a KMO value of 0.93 and a significant Bartlett’s sphericity test (χ^2^ = 2785.34, *p* = 0.004). Four factors were extracted, each comprising a minimum of three items. The factor loadings did not intersect, and all exceeded 0.40. The cumulative variance contribution amounted to 63.58% (> 40%). Refer to Table 4 for detailed information. The results of the CFA indicated favorable model fit indices: χ^2^/ν = 1.40 (<3); RMSEA = 0.05 (<0.08), RMSEA 90% confidence interval was 0.03–0.06; RMSR = 0.03 (<0.05); CFI = 0.98, TLI = 0.97 and IFI = 0.97. Additional details can be found in Figure 1.

**Table 4:** Factor loading of each item in the Chinese version of the scale.

Item	Factor 1	Factor 2	Factor 3	Factor 4
1. I am emotionally strong	0.825			
2. Spending energy on my family (such as taking care of household chores, attending to my child’s study) makes me feel good	0.672			
3. I can maintain a positive outlook toward the future during difficult times	0.797			
4. I believe that my family can become stronger through facing and overcoming difficulties	0.715			
5. When I feel bad, I believe that better times are coming	0.535			
6. I have future dreams that keep me going and motivate me	0.659			
7. In my life, the presence of my child’s cancer has made me a more understanding and tolerant person	0.506			
8. I trust my strengths to overcome the difficulties	0.623			
9. My belief in myself gives me strength to overcome difficult times	0.712			
10. I do not give up easily in the face of difficulties	0.641			
11. I use humor (jokes, quips, etc.) to overcome difficult times	0.543			
12. I can easily express my emotions (joy, pain, anger, fear) within my family	0.638			
13. In my life, the appearance of my child’s cancer has turned me into an anxious person		0.845		
14. I experience sleep problems on the nights before my child undergoing treatments such as chemotherapy and radiation therapy		0.799		
15. I have not slept as well since my child was diagnosed with cancer		0.764		
16. There are times when I feel emotionally exhausted		0.768		
17. I used to be more hopeful about life before my child was diagnosed with cancer		0.536		
18. I know where to seek medical help for my child’s cancer symptoms such as fever, pain, sleep disturbances			0.575	
19. I feel valued by my friends and family			0.686	
20. I can get support from my loved ones when I need it			0.637	
21. I can easily access healthcare services			0.628	
22. I spend too much time researching topics such as alternative cancer treatments, experiences, opportunities, etc. during the day				0.845
23. I cannot spend as much time as before on looking after myself, such as doing regular makeup, going to the hairdresser, shaving				0.808
24. I experience physical problems such as lower back, neck, and shoulder pain due to the stress I am experiencing				0.741

### 3.6 Internal structure validity analysis

The AVE values for the four factors ranged from 0.65 to 0.78, and the CR values ranged from 0.90 to 0.96. The square roots of the AVE values ranged from 0.81 to 0.88, all surpassing the correlation coefficients of the relevant components. For more detailed information, please refer to Table 5.

**Table 5:** Convergent and discriminant validity of the scale.

Factors	AVE	CR	Correlation between factors			
			Factor 1	Factor 2	Factor 3	Factor 4
Factor 1	0.670	0.960	0.818^a^			
Factor 2	0.649	0.902	0.471	0.806^a^		
Factor 3	0.699	0.903	0.669	0.443	0.836^a^	
Factor 4	0.780	0.914	0.555	0.539	0.541	0.883^a^

a Represents the square root of the AVE value.

### 3.7 Reliability analysis

The results of the reliability analysis indicated that the Cronbach’s α coefficient of the scale was 0.95, with respective coefficients for the four dimensions of 0.95, 0.90, 0.88, and 0.91. The total split-half reliability was 0.82, with corresponding coefficients for the four dimensions of 0.94, 0.90, 0.90, and 0.91. Furthermore, the total retest reliability was 0.95, with coefficients for the four dimensions of 0.93, 0.90, 0.91, and 0.95, respectively.

## 4 Discussion

### 4.1 The meanings and application values of the Chinese version of RSP-CC

Compared with family caregivers of children with other common diseases, parents of children with cancer experience greater caregiver burdens and emotional stress (Palacio et al., 2020). The presence of childhood cancer and its accompanying challenges can have significant implications for the physiological and mental health of parents (Shi et al., 2017; Bajjani-Gebara et al., 2019). Resilience, as a defensive strategy, directly impacts an individual’s self-worth, overall contentment, and sense of wellness, aiding in the preservation of both physical and mental health (Merino-Godoy et al., 2022). Studies have indicated that parents with strong resilience exhibit higher levels of hope and employ more proactive coping strategies when confronted with adversity (Rosenberg et al., 2019; Liu et al., 2021). Therefore, resilience training plays a vital role in enhancing parents’ ability to cope with challenges, highlighting the importance of understanding and assessing resilience among parents of children with cancer (Kunzler et al., 2020).

Currently, there remains a need for improved assessment tools to measure the resilience of these parents in China. We expect the introduction of a more dynamic, multidimensional, and tailored instrument to meet these assessment needs. The RSP-CC was developed based on the Resilience Model for Families of Children with Cancer, following an extensive literature review, expert consultation, and pilot study evaluation (Ye et al., 2017). This study was the first to translate the RSP-CC into Chinese and to validate its psychometric properties among these parents. It can serve as a critical tool for clinical staff in formulating specific interventions.

### 4.2 The psychometric properties of the Chinese version of RSP-CC

In the items analysis, the critical ratios (*t*-values) for each item of the scale ranged from 10.59 to 22.47, demonstrating a high level of item differentiation. The correlation coefficients between individual item scores and their corresponding factor scores ranged from 0.71 to 0.89, all exceeding 0.40, indicating extreme homogeneity across the overall scale.

Validity is the degree to which evidence and theory support the interpretations of test scores entailed by proposed uses of tests (AERA, APA, and NCME, 2014). In the original scale, the I-CVI values ranged from 0.88 to 1.00, and the S-CVI was 0.99 (Önal et al., 2023). In this study, the results from expert consultation showed I-CVI values ranging from 0.86 to 1.00, with an S⁃CVI of 0.97. Although these values were slightly lower than those reported for the original scale, they still exceeded the reference standards (I-CVI ≥ 0.78 and S-CVI ≥ 0.90) (Zamanzadeh et al., 2015), indicating good content validity for both versions. Furthermore, EFA extracted a four-factor structure with a cumulative variance contribution of 63.58%, slightly higher than the original scale’s 62.19%. The CFA further confirmed a well-matched four-factor model, indicating satisfactory internal validity. Finally, AVE values for the four factors ranged from 0.65 to 0.78, while CR values ranged from 0.90 to 0.96. The square roots of the AVE values were 0.81 to 0.88, surpassing the relevant components’ correlation coefficients, signifying strong convergent and discriminant validity.

Reliability refers to the ability of an instrument to consistently measure the thing or variable being measured (Koo and Li, 2016). In this study, the Cronbach′s α coefficient and split-half reliabilities for both the overall scale and its dimensions exceeded 0.7, indicating high internal consistency. The retest reliability after 2 weeks was 0.95, with dimensions ranging from 0.90 to 0.95, suggesting better temporal stability for the Chinese version of the RSP-CC.

### 4.3 Limitations of the current study

It is worth noting that there are still certain constraints in this study. First of all, we only examined the content validity and internal structural validity of the scale, omitting investigation into response validity and the construction of criterion validity. In order to further demonstrate the validity of the scale, it is worth considering the addition of more refined validity validation methods. Secondly, predictive validity was not explored, nor was the correlation of the scale’s total score with other potentially relevant measurement variables. Finally, potential biases arising from using the same data source for both exploratory factor analysis (EFA) and confirmatory factor analysis (CFA) were not addressed, potentially introducing method bias and selection bias.

### 4.4 Implications for future research

In future studies, it is recommended that researchers in related fields undertake further investigations into the structural validity of the Chinese version of the RSP-CC, and response validity and criterion validity should be incorporated into existing studies to elucidate the significance of the scale scores.

Secondly, exploring correlations between the scale scores and theoretically relevant measurement variables is crucial. For instance, examining predictive relationships between resilience and key variables such as anxiety, depression (van Gils et al., 2022), self-efficacy (Sousa et al., 2023), and coping styles (Koutelekos et al., 2023) could yield valuable insights. An innovative approach could involve exploring the transformation of ordinal scores into linear, equal-interval values using a logistic test model with Rasch methods, thereby demonstrating hierarchical relations among components in the parent caregiver response structure.

Furthermore, to mitigate method bias, future research could provide evidence of known group differences and employ a criterion approach to validity using the obtained parent score distribution. Specifically, researchers should endeavor to establish cross-modal correlations validation studies that validate differences in resilience scores between parents against relevant qualitative markers. These criterion data could be obtained from parent interviews or observer scores. Additionally, consideration of biological and neurological markers is warranted.

Lastly, to address the selection bias resulting from using the same data sources for both exploratory factor analysis (EFA) and confirmatory factor analysis (CFA), future researchers should implement resampling or bootstrapping methods for data collection.

## 5 Conclusion

After rigorous translation and verification, the RSP-CC was adapted in China, demonstrating favorable psychometric characteristics. It stands as an effective instrument for parents of children diagnosed with cancer in China. Additionally, this scale could serve as a crucial tool for clinical staff in formulating specific interventions.

## Data availability statement

The data collected in this investigation can be accessed by contacting the corresponding author upon a reasonable request. Requests to access these datasets should be directed to HC, 1319447367@qq.com.

## Ethics statement

The studies involving humans were approved by the Ethics Committee of Jinzhou Medical University (Ethics Approval No. JZMULL2023129). The studies were conducted in accordance with the local legislation and institutional requirements. Written informed consent for participation in this study was provided by the participants’ legal guardians/next of kin.

## Author contributions

GZ: Conceptualization, Data curation, Formal analysis, Investigation, Methodology, Project administration, Software, Supervision, Validation, Writing – original draft, Writing – review & editing. ZD: Formal analysis, Investigation, Methodology, Software, Validation, Writing – review & editing. XL: Investigation, Methodology, Supervision, Validation, Writing – review & editing. YQ: Investigation, Methodology, Project administration, Supervision, Validation, Writing – review & editing. XZ: Conceptualization, Formal analysis, Investigation, Methodology, Validation, Writing – review & editing. HC: Formal analysis, Funding acquisition, Methodology, Resources, Supervision, Validation, Visualization, Writing – original draft, Writing – review & editing.
